# Inhibitory Effects of Gymnema (*Gymnema sylvestre*) Leaves on Tumour Promotion in Two-Stage Mouse Skin Carcinogenesis

**DOI:** 10.1155/2014/328684

**Published:** 2014-03-06

**Authors:** Ken Yasukawa, Sakiko Okuda, Yasuhito Nobushi

**Affiliations:** School of Pharmacy, Nihon University, 7-7-1 Narashinodai, Funabashi, Chiba 274-8555, Japan

## Abstract

Ethanol extracts of gymnema (*Gymnema sylvestre*) leaves exhibited marked antitumour-promoting activity in an *in vivo* two-stage carcinogenesis test in mice using 7,12-dimethylbenz[*a*]anthracene as an initiator and 12-*O*-tetradecanoylphorbol-13-acetate (TPA) as a promoter. From the active fraction of the ethanol extract of the gymnema leaves, three triterpenoids were isolated and identified. These compounds were evaluated for their inhibitory effects on TPA-induced inflammation (1 µg/ear) in mice. The tested compounds showed marked anti-inflammatory effects, with a 50% inhibitory dose of 50–555 nmol/ear.

## 1. Introduction

The chemoprevention of cancer is an urgent priority in the field of public health. A method of prevention that acts at the promotion stage of carcinogenesis is most desirable, as such a method could be applied even after exposure to tumour-promotion agents, which in many cases is unavoidable in daily life. Many tumour promoters have inflammatory activity [1]. A correlation between the inhibitory effects against 12-*O*-tetradecanoylphorbol-13-acetate (TPA)-induced ear inflammation and inhibition of TPA-induced tumour promotion in a two-stage carcinogenesis experiment was observed in mice [2]. To screen for new inhibitors as chemopreventive agents, we intentionally selected natural compounds.

In the course of our studies on bioactive components from natural medicines, we found that the ethanol extracts of gymnema (*Gymnema sylvestre*; Asclepiadaceae) leaves showed inhibitory effects on TPA-induced inflammatory ear oedema in mice. Gymnema is a perennial herbaceous plant native to southern and central India and Sri Lanka. Gymnema has been used externally for diabetes [3, 4]. In chemical studies of gymnema leaves, the isolation and structural determination of flavonoids [5, 6], triterpenes [7, 8], and triterpene saponins [9–22] have been reported. With regard to biological activities, antiarthritic action [23] and inhibition of lipid absorption [24, 25] have been reported.

In the present study, ethanol extracts of gymnema leaves were found to inhibit TPA-induced tumour promotion during two-stage carcinogenesis in mouse skin. Three triterpenoids were isolated from ethanol extracts of gymnema leaves for inhibitory activity against TPA-induced inflammatory ear oedema in mice. The 50% inhibitory doses of these compounds for TPA-induced inflammatory ear oedema were 55–555 nmol/ear. Of the total assayed triterpenoids, 28-acetyl-21-tigloylgymnemagenin (**3**) showed similar activity as hydrocortisone, a steroidal anti-inflammatory drug, and gymnemagenin (**2**) and gymnemic acid III (**4**) showed greater suppression than indomethacin, a nonsteroidal anti-inflammatory drug.

## 2. Materials and Methods

### 2.1. General Experimental Procedures

^1^H- and ^13^C-NMR spectra were measured with a JEOL LA-600 (^1^H, 600 MHz; ^13^C, 150 MHz) spectrometer, and chemical shifts are presented as values relative to tetramethylsilane as an internal standard. Mass spectra were measured with a JEOL JMS-GC mate spectrometer at an ionization voltage of 70 eV. HPLC was performed on a C_18_ silica column (Cosmosil Cholester 10 id × 250 mm, Kyoto, Japan).

### 2.2. Chemicals

TPA was purchased from Chemicals for Cancer Research, Inc. (Eden Prairie, MN). 7,12-Dimethylbenz[*a*]anthracene, indomethacin, and hydrocortisone were obtained from Sigma Chemical Co. (St. Louis, MO). Acetone, chloroform, and methanol were obtained from Tokyo Kasei Kogyo Co., Ltd. (Tokyo, Japan).

### 2.3. Materials

Ethanol extracts of gymnema (*Gymnema sylvestre *R. Br.) leaves were obtained from Tokiwa Phytochemical Institute in April 2008. Voucher specimens “SM0802” were deposited at the Laboratory of Self Medication, School of Pharmacy, Nihon University.

### 2.4. Extraction and Isolation

Ethanol extracts (50 g) of gymnema leaves were partitioned within EtOAc-H_2_O (1 : 1) to yield an EtOAc extract (9.83 g). EtOAc extracts were partitioned within  *n*-hexane-MeOH-H_2_O (19 : 19 : 2), which afforded *n*-hexane (685 mg) and MeOH-H_2_O (9.10 g) extracts, respectively. The H_2_O solution was partitioned within  *n*-BuOH-H_2_O (1 : 1), yielding an *n*-BuOH extract (19.5 g) and an H_2_O extract (20.2 g), respectively.

MeOH-H_2_O extracts (9 g) were subjected to column chromatography (CC) on Silica gel 60 (E.Merck, Germany) using CHCl_3_-MeOH (100 : 0→0 : 100) to obtain five fractions: fraction 1 (81.6 mg), fraction 2 (758 mg), fraction 3 (1.14 g), fraction 4 (4.09 g), and fraction 5 (2.84 g). Fraction 3 (1.0 g) was further separated on ODS 75 C-_18_ (Nacalai Tesque, Kyoto, Japan) using MeOH : H_2_O (20 : 80→100 : 0) to obtain nine fractions: 3-1 (77.4 mg), 3-2 (15.9 mg), 3-3 (58.8 mg), 3-4 (2.57 mg), 3-5 (24.1 mg), 3-6 (7.48 mg), 3-7 (18.1 mg), 3-8 (31.8 mg), and 3-9 (699 mg). Fraction 3-3 (50.0 mg) was purified by reversed-phase preparative HPLC to isolate **1** (5.3 mg). Fractions 3-5 (20.0 mg) and 3-7 (13.0 mg) were treated by the same method to isolate **2** (5.5 mg) and **3** (7.6 mg), respectively. Fraction 4 (4.0 g) was separated on ODS 75 C-_18_ using MeOH-H_2_O (50 : 50→100 : 0) to obtain three fractions: 4-1 (215 mg), 4-2 (2.67 g), and 4-3 (1.09 g). Fraction 4-2 (2.5 g) was separated by Silica gel 60 using CHCl_3_-MeOH : H_2_O (90 : 10 : 1→0 : 100 : 0) to obtain four fractions: 4-2-1 (388 mg), 4-2-2 (255 mg), 4-2-3 (641 mg), and 4-2-4 (1.03 g). Fraction 4-2-1 was then purified by HPLC to isolate **4** (4.8 mg).

### 2.5. Identification

Identification of compounds **1**, **2**, and **4** was performed by spectral comparison with literature data (Figure 2). Compound **1** was identified as phenethyl *β*-d-glucoside [26]. Compounds **2** and **4** were identified as gymnemagenin (**2**) and gymnemic acid III (**4**) [14]. Compound **3**, a pale yellow amorphous powder, [*α*]d + 37.8° (*c* = 1.0, MeOH), possessed the molecular formula C_37_H_58_O_8_, HR-FAB-MS (positive mode): *m/z* 631.42124 [M + H]^+^ (calcd. 631.42097), suggesting that **3** was composed of **2**, acetic acid, and tiglic acid. By comparison of **3** with **2** on ^13^C-NMR spectra, two acylation shifts were observed at the C-21 (position) [+1.9 ppm (C-21)] and the C-28 (position) [+4.1 ppm (C-28)] [17]. In addition, the chemical shifts of **3** accorded with that of the aglycone moiety of gymnemic acid I in ^13^C-NMR spectral data [9, 13, 17]. These results suggest that **3** is 28-acetyl-21-tigloylgymnemagenin (**3**) (^13^C-NMR *δ* (in pridine-*d*_5_, 150 MHz): 171.2 (Ac-1), 168.4 (Tig-1), 141.7 (C-13), 137.1 (Tig-3), 129.9 (Tig-2), 124.3 (C-12), 79.2 (C-3), 73.7 (C-21), 72.0 (C-22), 68.3 (C-16), 67.9 (C-23), 62.7 (C-28), 48.9 (C-5), 47.6 (C-9), 46.2 (C-17), 46.2 (C-19), 43.3 (C-4), 43.1 (C-14), 42.9 (C-18), 40.6 (C-8), 39.4 (C-1), 37.4 (C-10), 37.0 (C-20), 36.7 (C-15), 33.0 (C-7), 29.7 (C-29), 28.0 (C-27), 27.8 (C-2), 24.4 (C-11), 21.1 (Ac-2), 20.1 (C-30), 18.8 (C-6), 17.5 (C-26), 16.5 (C-25), 14.5 (Tig-4), 13.4 (C-24), 12.8 (Tig-5)). Full details of the identification, as well as the spectral data, are available on request from the corresponding author.

### 2.6. Animals

Experiments were performed in accordance with the Guidelines of the Institutional Animal Care and Use Committee of the College of Pharmacy, Nihon University, Chiba, Japan. Female ICR mice (age: 7 weeks) were purchased from Japan SLC Inc. (Shizuoka, Japan) and were housed in an air-conditioned specific pathogen-free room (24 ± 2°C) lit from 08:00 to 20:00. Food and water were available *ad libitum*.

### 2.7. TPA-Induced Inflammation Assay in Mice

TPA (1 *μ*g) dissolved in acetone (20 *μ*L) was applied to the right ear of ICR mice by means of a micropipette. A volume of 10 *μ*L was delivered to both the inner and outer surfaces of the ear. The sample (0.02–1.0 mg/ear) or vehicle, methanol-chloroform-water (2 : 1 : 1; 20 *μ*L) or methanol-chloroform (1 : 1; 20 *μ*L), as a control, was applied topically about 30 min before TPA treatment. For ear thickness determination, a pocket thickness gauge (Mitsutoyo Co. Ltd., Tokyo, Japan) with a range of 0–9 mm (graduated at 0.01-mm intervals and modified so that the contact surface area was increased, thus reducing tension) was applied to the tip of the ear.

Ear thickness was determined before TPA treatment (*a*).

Oedema was measured at 6 h after TPA treatment (*b*: TPA with vehicle; *b*′: TPA with sample). The following values were then calculated:

 Oedema A: oedema induced by TPA with vehicle (*b* − *a*).
 Oedema B: oedema induced by TPA plus sample (*b*′ − *a*).
 Inhibitory ratio (%) = [(oedema A − oedema B)/oedema A] × 100.

Each value was the mean of individual determinations from four mice.

### 2.8. Two-Stage Carcinogenesis Experiment

The backs of mice (age: 7 weeks) were shaved with electric clippers. Initiation was accomplished by a single topical application of 50 *μ*g of DMBA. Promotion with 1 *μ*g TPA, applied twice weekly, was started 1 week after initiation. Ethanol extracts of gymnema leaves (1.0 mg/mouse) or vehicle, acetone-dimethylsulfoxide-water (8 : 1 : 1; 100 *μ*L), was applied topically 30 min before each TPA treatment. DMBA and TPA were dissolved in acetone and applied to the shaved area in a volume of 100 *μ*L using a micropipette. The back of each animal was shaved once a week to remove hair. The number and diameter of skin tumours were measured every week, and the experiment was continued for 20 weeks. Experimental and control groups each consisted of 15 mice.

### 2.9. Statistical Analysis

The 50% inhibitory dose (ID_50_) values and their 95% confidence intervals (95% CI) were obtained by nonlinear regression using the GraphPad program 5.0 (Intuitive Software for Science, San Diego, CA). Differences between experimental groups were compared by Student's *t*-test and Mann-Whitney *U* exact test.

## 3. Results and Discussion

**Figure 1:**
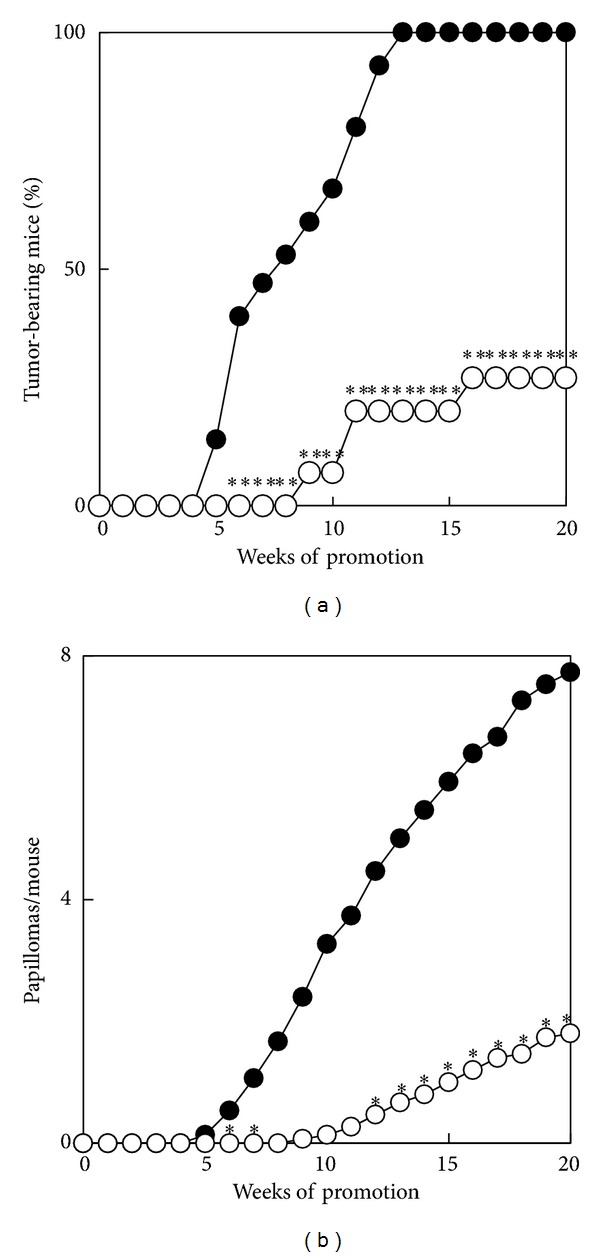
Inhibitory effects of ethanol extracts of gymnema leaves on tumour promotion of skin papillomas by TPA in DMBA-initiated mice. From 1 week after initiation with a single topical application of 50 *μ*g of DMBA, 1 *μ*g of TPA was applied twice weekly. Topical application of ethanol extract (1 mg) and vehicle was performed 30 min before each TPA treatment. Data are expressed as the percentage of mice bearing papillomas (a) and as the average number of papillomas per mouse (b). ●, +TPA with vehicle alone; ○, +TPA with ethanol extract of gymnema leaves. The treated group was determined to be statistically different from the control group by Mann-Whitney  *U*  exact test (a) and by Student's *t*-test (b). *P < 0.05 and **P < 0.01.

**Figure 2:**
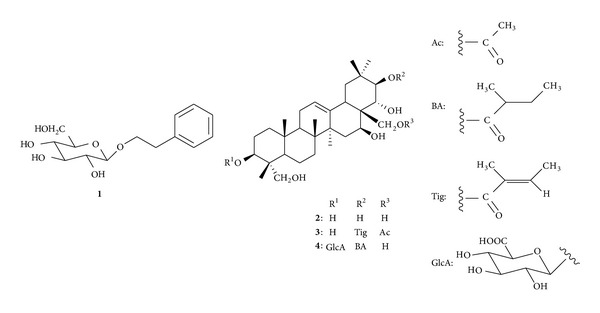
Chemical structures of components from gymnema leaves.

As can be seen in Table 1, extracts from gymnema leaves inhibited TPA-induced inflammation in mice. The inhibitory effects of the ethanol extract of gymnema leaves in a two-stage carcinogenesis test on mouse skin using DMBA as an initiator and TPA as a tumour promoter were then investigated.  Figure 1(a) illustrates the time course of skin tumour formation in the groups treated with DMBA plus TPA, with or without the ethanol extract of gymnema leaves. The first tumour appeared at week 5 in the group treated with DMBA plus TPA and all 15 mice had tumours at week 13. In the group treated with DMBA plus TPA and ethanol extract of gymnema leaves, the first tumour appeared at week 9. The percentage of tumour-bearing mice treated with DMBA plus TPA and ethanol extract of gymnema leaves was 27% at week 20.  Figure 1(b) shows the average number of tumours per mouse. The group treated with DMBA plus TPA produced 7.7 tumours per mouse at week 20; the group treated with DMBA plus TPA and ethanol extract of gymnema leaves had 1.8 tumours per mouse. Treatment with ethanol extract of gymnema leaves caused a 77% reduction in the average number of tumours per mouse at week 20.

Active components were then isolated from the ethanol extract of gymnema leaves. The isolated compounds showed inhibitory activity against TPA-induced ear inflammatory oedema. As can be seen in Table 2, the ID_50_ values of **2**–**4** against TPA-induced inflammation were 49.7–555 nmol/ear, respectively.

This is the first report to find that ethanol extracts of gymnema leaves inhibit tumour promotion by TPA following initiation with DMBA in ICR mouse skin. Furthermore, the active constituents were identified as three triterpenes from ethanol extracts of gymnema leaves. This is the first report of phenethyl glucoside in the genus *Gymnema* and the first report of 28-acetyl-21-tigyolgymnemagenin in nature.

These results demonstrate the efficacy to two triterpenes and triterpene glycosides in the components of gymnema leaves. In our study, we reported that numerous triterpenes and their glycosides are effective for preventing cancer [27, 28]. Therefore, we inferred that triterpenes and their glycosides in gymnema leaves are the active components.

## Figures and Tables

**Table 1:** Inhibitory effects of gymnema leaves on TPA-induced inflammatory ear oedema.

Sample	I.R.
EtOH extract (1.0 mg/ear)	82**
*n*-Hexane extract of EtOH extract (1.0 mg/ear)	75**
MeOH-H_2_O extract of EtOH extract (1.0 mg/ear)	89**
*n*-BuOH extract of EtOH extract (1.0 mg/ear)	43**
H_2_O extract of EtOH extract (1.0 mg/ear)	9
Fraction 1 from MeOH-H_2_O extract of EtOH extract (0.5 mg/ear)	29*
Fraction 2 from MeOH-H_2_O extract of EtOH extract (0.5 mg/ear)	45**
Fraction 3 from MeOH-H_2_O extract of EtOH extract (0.5 mg/ear)	62**
Fraction 4 from MeOH-H_2_O extract of EtOH extract (0.5 mg/ear)	89**
Fraction 5 from MeOH-H_2_O extract of EtOH extract (0.5 mg/ear)	41**

I.R.: inhibitory ratio. *P < 0.05 and **P < 0.01.

**Table 2:** Inhibitory effects of components from gymnema leaves on TPA-induced inflammatory ear oedema.

Component	ID50	95% CI
	(nmol/ear)	
Gymnemagenin (**2**)	555	417–739
28-Acetyl-21-tigloylgymnemagenin (**3**)	49.7	38.3–64.7
Gymnemic acid III (**4**)	212	160–281

Indomethacin^a^	908	755–1092
Hydrocortisone^a^	69.1	64.3–75.4

ID_50_: 50% inhibitory dose. 95% CI: 95% confidence intervals. ^a^Reference compound.
